# Comparative efficacy of exercise training modes on systemic metabolic health in adults with overweight and obesity: a network meta-analysis of randomized controlled trials

**DOI:** 10.3389/fendo.2023.1294362

**Published:** 2024-01-16

**Authors:** Huiying Wang, Ruitang Cheng, Lijun Xie, Fang Hu

**Affiliations:** National Clinical Research Center for Metabolic Diseases, Key Laboratory of Diabetes Immunology, Ministry of Education, Department of Metabolism and Endocrinology, The Second Xiangya Hospital of Central South University, Changsha, Hunan, China

**Keywords:** exercise training, obesity, metabolism, network meta-analysis, HIIT

## Abstract

**Objective:**

This network meta-analysis (NMA) was conducted to compare and rank the effects of training interventions including aerobic exercise (AE), resistance training (RT), combined aerobic and resistance training (CT), and high-intensity interval training (HIIT) on vital metabolic indicators in adults with overweight and obesity.

**Methods:**

PubMed, Cochrane, Embase, and Web of Science were searched from 1990 to February 2023. Articles were included if they described randomized controlled trials (RCTs) examining the effects of exercise training on anthropometry parameters, lipid profiles, glucose metabolism, blood pressure, and cardiorespiratory fitness in adults with overweight and obesity. Weighted mean difference with 95% CI was calculated.

**Results:**

A total of 28 studies with 1,620 patients were included. Results revealed that AE exerts best effects on weight loss (−2.35 [−4.05, −0.64]) and body mass index (−0.9 [−1.38, −0.42]), while HIIT is the most effective in reducing waist circumference (−5.93 [10.71, −1.15]), percentage body fat (−3.93 [−5.73, −2.12]), serum triglycerides (−20.55 [−37.20, −3.91]), and fasting blood glucose (−14.31 [−22.47, −6.16]) and improving VO_2_ max (7.41 [4.37, 10.45]). However, no significant benefit was observed in terms of total cholesterol and blood pressure.

**Conclusions:**

AE is the optimal exercise type for reducing body weight and BMI, while HIIT exerts the most beneficial effects on improving body composition, cardiorespiratory fitness, and metabolic abnormalities in adults with overweight and obesity.

**Systematic review registration:**

https://www.crd.york.ac.uk/prospero/display_record.php?ID=CRD42023444322, identifier CRD42023444322.

## 1 Introduction

Obesity is predicted to affect nearly 20% of people around the world and is causing a huge burden as a noncommunicable disease (1). Given the enormous personal and medical costs of overweight and obesity, it is urgent for public health policymakers to identify effective interventions.

Owing to the lack of safe and effective drugs for the treatment of obesity, the main interventions for obesity are still diet control and exercise. The main forms of exercise are aerobic exercise (AE), resistance training (RT), combined aerobic and resistance training (CT), and high-intensity interval training (HIIT) (2). According to the recommendation of guidelines of exercise for people with obesity, moderate-intensity AE for at least 300 min per week (3) was the preferred choice. However, the effects of different exercise modalities on body composition, metabolic health, and cardiovascular health in people with obesity were inconsistent. A meta-analysis reported that AE is beneficial for reducing body weight and fat mass, while RT is effective in reducing fat mass (4). Another meta-analysis showed that HIIT was slightly more effective than AE in improving VO_2_ max (5). Moreover, a meta-analysis of adolescents with obesity has shown that compared with AE alone, CT resulted in greater reductions in LDL levels (6).

Despite the fact that it is well known that exercise is effective in reducing body weight and fat mass and promoting cardiovascular metabolism, a potential drawback is the large weekly time consumption, which totals approximately 300 to 400 min per week. Thus, in recent years, HIIT is being increasingly recommended as an alternative option mainly because it has high efficiency and is time-saving, as lack of time is the main obstacle that prevents people from exercising (7). There is increasingly strong evidence showing that HIIT has better effects on a series of health indicators in both healthy and chronically ill people than continuous moderate-intensity training (8–10). However, in terms of the effects of exercise training on people with overweight and obesity, most studies are pairwise meta-analyses that cannot rank the effectiveness of different trainings.

Different from a conventional meta-analysis, a network meta-analysis (NMA) is a powerful tool for comparing the effectiveness of different interventions as it can combine direct and indirect evidence and allow for the ranking of treatments (11). However, the current available NMA focused only on the effects among AE, RT, and CT, without considering the HIIT. More importantly, it is unclear which type of exercise is most effective for the different metabolic abnormalities in people with overweight and obesity. Therefore, the aims of this NMA were to comprehensively evaluate and rank the effectiveness of exercise across the vital metabolic indicators including anthropometry, glucose homeostasis, lipid profile, and cardiovascular fitness in adults with overweight and obesity.

## 2 Materials and methods

This systematic review and NMA follows the Preferred Reporting Items for Systematic Reviews and Meta-Analyses for Network Meta-Analyses (PRISMA-NMA) (12) and is registered in the PROSPERO database (Registration number: CRD42023444322).

### 2.1 Literature search strategy

Four electronic databases (PubMed, Cochrane, Embase, and Web of Science) were searched from 1900 to February 2023. The search strategy was based on the PICOS tool: (P) Population: individuals with overweight or obesity; (I) Intervention: exercise; (C) Comparator: no exercise or other exercise modality; (O) Outcomes: body composition, lipid metabolism, glucose metabolism, blood pressure, and VO_2_ max; (S) Study type: randomized controlled trial (RCT). A complete list of the search terms is available in the Supplementary Materials section (**Supplementary Table 1**).

### 2.2 Inclusion and exclusion criteria

Studies were considered eligible for inclusion if the following criteria were met: (1) RCT must be published in English. (2) Participants must be adult individuals with overweight or obesity (BMI ≥25 kg/m^2^). (3) Studies employed an intervention of AE, RT, CT, or HIIT for at least 4 weeks. The definition of exercise interventions is shown in **Supplementary Table 2**. (4) Outcome of interest included anthropometry (body weight [BW], body mass index [BMI], waist circumference [WC], and percentage body fat [%BF]), lipid metabolism (triglycerides [TG], total cholesterol [TC], high-density lipoprotein [HDL], and low-density lipoprotein [LDL]), glucose metabolism (fasting blood glucose [FBG], fasting blood insulin [FINS], and homeostatic model assessment for insulin resistance [HOMA-IR]), and cardiovascular function (systolic blood pressure [SBP], diastolic blood pressure [DBP], and VO_2_ max).

The exclusion criteria were as follows: (1) The intervention was combined with diet control. (2) The subjects were reported to be taking medication or had other noncommunicable diseases, such as cancer, hypertension, and diabetes. (3) Articles that did not include the outcome of interest. (4) Conference abstracts, case reports, and dissertations were excluded. (5) Duplications of the searched studies.

Abstracts and full texts were screened by two authors independently, and any uncertainty was discussed among authors.

### 2.3 Data extraction

Data were extracted by two authors independently. The following characteristics were extracted: (1) first author; (2) publication year; (3) country; (4) sample size; (5) mean age; (6) characteristics of the participants; (7) outcome measures including BW [kg], BMI [kg/m^2^], WC [cm], %BF [percentage], TG [mg/dL], TC [mg/dL], LDL [mg/dL], HDL [mg/dL], FBG [mg/dL], FINS [μU/mL], HOMA-IR, SBP [mmHg], DBP [mmHg], and VO_2_ max [mL/kg·min]; and (8) description of the exercise intervention (type, intensity, duration, and frequency).

### 2.4 Assessment of study quality

We used the Cochrane Risk of Bias Tool (13) to assess the risk of bias (ROB) of the included studies independently. The following domains were considered: (1) randomization process, (2) treatment allocation concealment, (3) blinding of participants and researchers, (4) blinding of outcome assessment, (5) incomplete outcome data, (6) selective reporting, and (7) other bias.

### 2.5 Statistical analysis

We use weighted mean difference and 95% confidence interval (CI) to report continuous variables. Review Manager 5.3 was used for the traditional pairwise meta-analysis. Statistical heterogeneity was evaluated using the (*I* ^2^) statistic: *I* ^2^ > 50% was considered high heterogeneity and random-effects model was employed; otherwise, the fixed-effects model was adopted. For the NMA, Stata16.0. software package “mvmeta” and “network” was used to analyze indirect comparisons between different interventions.

The network diagram was used to present the relationship between exercise interventions. The node-splitting method and loop inconsistency test were performed to test inconsistency. *p* > 0.05 indicates that there is no significant inconsistency and the consistency model is chosen; otherwise, the inconsistency model is employed (14, 15).

The effectiveness of different interventions was ranked by using the surface under the cumulative ranking probability diagram (SUCRA) (16). The value of SUCRA is higher, and the effects of interventions are better. League tables were used to present the pair-to-pair comparisons between the exercise interventions.

Furthermore, a comparison-adjusted funnel plot was used to assess publication bias. To explore the heterogeneity, subgroup analyses were performed in pairwise meta-analyses, including different duration of exercise (4–8 weeks as short-term duration and >9 weeks as long-term duration) and mean baseline BMI (25–30 kg/m^2^ as individuals with overweight and ≥30 kg/m^2^ as individuals with obesity).

## 3 Results

### 3.1 Literature selection

**Figure 1:**
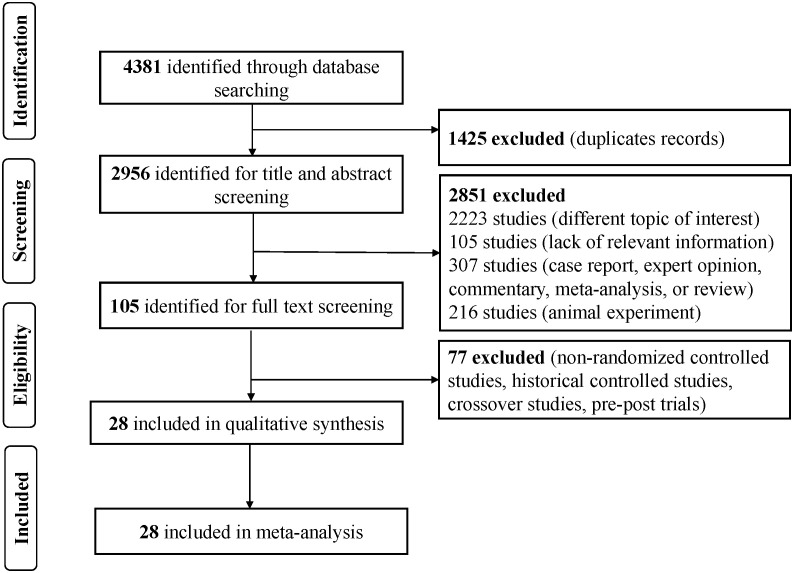
Schema of the search strategy.

According to the search strategy, 4,381 studies were identified in those four databases. After 1,425 duplicates were removed, 2,956 articles were left to screen the titles and abstracts. After reading the full text of 105 studies, we included 28 RCTs for the NMA. The flowchart of the search and selection process is presented in **Figure 1**.

### 3.2 Characteristics of included studies

**Table 1** shows the clinical and demographic characteristics of the 28 included studies (17–44). A total of 1,620 participants were enrolled in the NMA; sample size ranged from 21 to 349. As for gender, 15 studies recruited women only and 8 studies recruited men only, and 5 studies included both men and women. Of the total, 75% participants were women. With regard to exercise categories, participants were included in the AE (*n* = 757), RT (*n* = 108), CT (*n* = 107), and HIIT (*n* = 111) groups, respectively. The duration of the training ranged from 4 weeks to 48 weeks (mostly 12 weeks). The number of weekly sessions was mostly three times a week (*n* = 20).

**Table 1:** Study characteristics.

Study	Country	Sample	Mean age	Exercise category	Intervening measure and intensity	Intervention duration	Outcome measures reported
		N/M/F	(SD)				
Sadegh 2022	Iran	11/11/0	27–32	CON	No exercise	8 weeks	BW, BMI, FBG, FINS, HOMA-IR, TC, LDL, HDL, TG, %BF, VO_2_ max
		32/32/0		HIIT	5×2-min, 85%–95% of VO_2_peak, 1-min, 13-s inactive recovery		
Mehdi 2022	Iran	20/20/0	45–55	CON	No exercise	12 weeks	BW, BMI, WC, FBG, FINS, HOMA-IR, VO_2_ max
		22/22/0		AE	70%–75% MHR		
Elaheh 2022	Iran	8/8/0	23.12 ± 0.55	CON	No exercise	8 weeks	FBG, FINS, HOMA-IR
		9/9/0	22.62 ± 0.73	AE	50%–80% MHR		
		9/9/0	22.40 ± 0.65	RT	30%–70% 1RM		
Bik 2021	China	10/0/10	19.4 ± 0.5	CON	No exercise	12 weeks	BW, BMI, FBG, FINS, HOMA-IR, %BF
		21/0/21	19.8 ± 0.9	AE	Descending stair or ascending stair		
Małgorzata 2021	Poland	44/0/44	55.0 ± 7.0	AE	50%–70% MHR	12 weeks	BW, BMI, WC
		41/0/41		CT	AE: 50%–70% MHR RT: 20 min, 50%–60% of one-repetition maximum		
Carolyn 2021	US	11/7/4	53.1 ± 6.4	AE	65%–80% peak VO_2_	24 weeks	BW, BMI, WC, SBP, DBP, VO_2_ max
		10/3/7	47.0 ± 12.3	RT	70%–85% 1RM		
Alberto 2021	Spain	10/0/10	56.9 ± 5.8	CON	No exercise	12 weeks	BW, BMI, FBG, FINS, HOMA-IR, TC, LDL, HDL, TG, %BF, VO_2_ max
		24/0/24	49.3 ± 7.6	AE	55%–75% HRR		
		13/0/13	58.7 ± 2.9	CT	AE: 55%–75% HRR, RT: 65% 1RM with 60–90 s of rest		
Samaneh 2020	Iran	9/0/9	44.2 ± 3.6	CON	No exercise	12 weeks	SBP, TC, LDL, TG
		10/0/10	42.8 ± 2.7	HIIT	4 min at 85%–95% MHR with 3 min breaks at 50%–60% MHR		
Tze Y 2020	Australia	30/0/30	53.2 ± 3.1	CON	No exercise	8 weeks	BW, BMI, SBP, DBP, FBG, TC, LDL, HDL
		30/0/30	53.9 ± 3.4	HIIT	20 min at 70% MHR with 5 min breaks		
Pamela 2020	South Africa	15/0/15	20–35	CON	No exercise	12 weeks	BW, BMI, WC, FBG, FINS, HOMA-IR, %BF, VO_2_ max
		20/0/20		CT	AE: 75%–80% HR peak, RT: 60%–70% HR peak		
Mehdi 2019	Iran	10/0/10	21.5 ± 2.4	CON	No exercise	8 weeks	BW, BMI, WC, VO_2_ max
		12/0/12	22.8 ± 2.4	AE	65% MHR		
		12/0/12	22.5 ± 2.7	RT	2–4 sets, 8–12 repetitions, with a 60–90 s break		
Paulo 2019	US	13/0/13	62.9 ± 8.8	CT	AE: 70% of MHR, RT: 70% of 1RM	12 weeks	BW, BMI, %BF
		13/0/13	62.3 ± 6.9	HIIT	60 s >80% MHR with 60 s Low-intensity exercise at 60% of MHR		
Kalyana 2018	India	64/30/34	20-45	CON	No exercise	12 weeks	BW, BMI, WC, FBG, TC, LDL, HDL, TG, %BF, VO_2_ max
		66/29/37		AE	40%–60% HRR		
Lyndsey 2018	USA	11/0/11	28.3 ± 6.1	AE	60%–70% MHR	16 weeks	BW, BMI, WC, SBP, DBP, FBG, FINS, HOMA-IR, TC, HDL, TG, %BF, VO_2_ max
		16/0/16	32.1 ± 7.0	HIIT	3 min at 60%–70% MHR, 1 min at 80%–90% MHR, with 5–10 min breaks		
Mahmoud 2018	Iran	10/10/0	39.1 ± 3.1	CON	No exercise	12 weeks	BW, WC, FBG, FINS, HOMA-IR, %BF, VO_2_ max
		12/12/0	40.0 ± 3.1	RT	40%–95% 1RM		
Nicholas 2018	South Africa	15/0/15	24.5 ± 0.9	CON	No exercise	12 weeks	BW, WC, TC, LDL, HDL VO_2_ max
		20/0/20	22.8 ± 0.7	CT	AE: 75%–80% HR peak, RT: 60%–70% HR peak		
Bladbjerg 2017	Denmark	12/12/0	31.0 ± 7.0	CON	No exercise	12 weeks	BW, BMI
		12/12/0	28.0 ± 5.0	AE	Intensity: unclear; endurance training		
Catherine 2017	USA	44/0/44	58.5 ± 5.2	CON	No exercise	48 weeks	BMI
		25/0/25		AE	Moderate-to-vigorous intensity aerobic exercise intervention		
Anne 2017	Denmark	16/9/7	35.0 ± 7.5	CON	No exercise	24 weeks	BW, BMI, VO_2_ max
		74/37/37	34.5 ± 7.1	AE	50%–70% VO_2_ peak endurance exercise		
Yunsuk 2016	USA	12	18–65	CON	No exercise	4 weeks	BW, BMI, %BF
		15		AE	70% MHR		
Nina 2015	Chicago	8/0/8	30.8 ± 9.0	CON	No exercise	8 weeks	BW, BMI, WC, SBP, DBP, FBG, TC, LDL, HDL, %BF
		10/0/10	30.3 ± 5.4	RT	85%–95% 10-RM		
Croymans 2014	USA	8/8/0	22.0 ± 1.2	CON	No exercise	12 weeks	BW, BMI, WC, SBP, DBP, %BF
		28/28/0	21.5 ± 3.9	RT	40%–60% 1RM		
Therese 2014	Northern Ireland	25/8/17	45.0 ± 7.4	CON	Light stretching exercises	24 weeks	BMI, WC, SBP, DBP, TC, LDL, HDL, TG, %BF
		52/11/41	15.0 ± 6.2	AE	10-min bouts of brisk walking		
Mahmoud 2014	Iran	11/11/0	38.9 ± 4.1	CON	No exercise	12 weeks	BW, WC, %BF, VO_2_ max
		12/12/0	40.4 ± 5.2	RT	40%–95% 1RM		
		10/10/0	39.6 ± 3.7	HIIT	4 min at 80%–90% MHR, 3-min jogging at 55%–65% MHR		
Neil 2012	America	86/0/86	57.1 ± 5.7	CON	No exercise	24 weeks	BW, BMI, WC, FBG, FINS, HOMA-IR, VO_2_ max
		78/0/78	57.2 ± 6.8	AE	HR: 50% VO_2_peak		
Benoit 2009	Canada	82/0/82	57.2 ± 6.1	CON	No exercise	24 weeks	BW, BMI, WC, SBP, DBP, FBG, FINS, HOMA-IR, TC, LDL, HDL, TG, VO_2_ max
		267/0/267	57.3 ± 6.6	AE	HR: 50% VO_2_ max		
Brixius 2008	Germany	6/6/0	52.6 ± 1.1	CON	No exercise	24 weeks	SBP, DBP
		14/14/0	58.8 ± 1.3	AE	pulse 2–4 mmol/L lactate		
Thomas 2006	America	15/0/15	38.0 ± 2.0	CON	No exercise	48 weeks	BW, BMI, SBP, DBP, FBG, TC, LDL, HDL, TG, %BF
		15/0/15	38.0 ± 1.0	RT	three sets of 8–10 repetitions		

CON, control group; AE, aerobic exercise; RT, resistance training; CT, aerobic combined resistance training; HIIT, high-intensity interval training.BW, body weight; BMI, body mass index; WC, waist circumference; %BF, percentage body fat; FBG, fasting blood glucose; FINS, fasting blood insulin; TG, triglycerides; HDL, high-density lipoprotein; LDL, low-density lipoprotein; MHR, maximum heart rate; RM, repetition maximum; HRR heart rate reserve.

### 3.3 Risk of bias and publication bias in the enrolled studies

The summary of the risk of bias assessment is shown in **Supplementary Figure 1**. In brief, 15 studies showed unclear ROB in random sequence generation, allocation concealment, and blinding of the participants and implementers. All studies showed low ROB in blinding of outcome assessment and selective reporting. Only one study showed risk of other biases. Overall, the quality of the included studies was considered moderate.

### 3.4 Network meta-analysis and subgroup analyses

Four measures of anthropometry and body composition (BW, BMI, WC, and %BF), three measures of lipid metabolism (TG, HDL, and LDL), three measures of glucose metabolism (FBG, FINS, and HOMA-IR), and one measure of cardiovascular function (VO_2_ max) were included in the NMA. **Table 2** illustrates the results of pair-to-pair comparisons between the exercise interventions. **Figure 2** illustrates NMA maps of studies examining the efficacy of exercise modes on 11 indicators. **Table 3** presents the rank of exercise interventions in order of effectiveness. The results of subgroup analyses are shown in **Supplementary Table 3**. The results of interval plot and cumulative ranking probability plots of NMA can be found in **Supplementary Figures 2**-**23**. The results of local inconsistency are displayed in **Supplementary Figures 24**-**34**. Funnel plot graphics are illustrated in **Supplementary Figures 35**-**45**.

**Table 2:** Matrix of the network meta-analysis results.

BW, kg				
HIIT				
−0.44 (−4.20, 3.32)	**AE**			
−0.90 (−4.97, 3.18)	−0.46 (−3.20, 2.29)	**CT**		
−2.46 (−6.49, 1.57)	−2.02 (−4.96, 0.92)	−1.56 (−5.06, 1.93)	**RT**	
**−2.79 (−6.25, 0.68)**	−2.35 (−4.05, −0.64)	−1.89 (−4.27, 0.49)	−0.33 (−2.95, 2.30)	**CON**
BMI, kg/m^2^				
AE				
−0.14 (−1.17, 0.88)	**CT**			
−0.43 (−1.96, 1.10)	−0.28 (−1.99, 1.42)	**HIIT**		
−1.19 (−2.14, −0.24)	−1.05 (−2.35, 0.26)	−0.76 (−2.48, 0.95)	**RT**	
−**0.90 (−1.38, −0.42)**	−0.76 (−1.73, 0.21)	−0.48 (−1.96, 1.01)	0.29 (−0.61, 1.18)	**CON**
WC, cm				
CT				
−0.01 (−5.32, 5.31)	**HIIT**			
−2.90 (−6.24, 0.45)	−2.89 (−7.71, 1.93)	**RT**		
−3.09 (−5.88, −0.29)	−3.08 (−7.94, 1.78)	−0.19 (−2.66, 2.28)	**AE**	
−**5.94 (−8.33**, −**3.55)**	−5.93 (−10.71, −1.15)	−3.04 (−5.42, −0.67)	−2.85 (−4.63, −1.08)	**CON**
%BF, %				
HIIT				
−1.25 (−3.69, 1.18)	**AE**			
−1.28 (−3.47, 0.90)	−0.03 (−2.54, 2.48)	**RT**		
−2.65 (−5.43, 0.13)	−1.40 (−4.55, 1.75)	−1.37 (−4.26, 1.52)	**CT**	
−**3.93 (−5.73, −2.12)**	−2.67 (−4.67, −0.67)	−2.64 (−4.23, −1.06)	−1.27 (−3.76, 1.21)	**CON**
FBG, mg/dL				
HIIT				
−9.19 (−17.37, −1.02)	**AE**			
−9.66 (−21.06, 1.75)	−0.46 (−8.93, 8.00)	**CT**		
−13.07 (−22.62, −3.52)	−3.87 (−9.89, 2.15)	−3.41 (−13.07, 6.25)	**RT**	
−**14.31 (−22.47, −6.16)**	−5.12 (−8.73, −1.51)	−4.66 (−12.90, 3.59)	−1.25 (−6.36, 3.86)	**CON**
FINS, μU/mL				
HIIT				
−3.17 (−7.88, 1.54)	**AE**			
−3.24 (−9.41, 2.93)	−0.07 (−4.41, 4.28)	**CT**		
−3.91 (−9.20, 1.39)	−0.74 (−3.83, 2.35)	−0.67 (−5.73, 4.39)	**RT**	
−**5.94 (−10.48, −1.40)**	−2.77 (−4.67, −0.87)	−2.70 (−7.01, 1.60)	−2.03 (−4.87, 0.81)	**CON**
HOMA-IR				
HIIT				
−2.10 (−4.37, 0.16)	**AE**			
−2.51 (−4.97, −0.04)	−0.40 (−1.73, 0.93)	**RT**		
−2.85 (−5.37, −0.33)	−0.75 (−2.26, 0.77)	−0.34 (−2.23, 1.54)	**CT**	
−**3.01 (−5.08, −0.94)**	−0.91 (−1.81, 0.00)	−0.50 (−1.84, 0.83)	−0.16 (−1.60, 1.28)	**CON**
TG, mg/dL				
HIIT				
−10.51 (−28.79, 7.77)	**AE**			
−11.70 (−41.69, 18.28)	−1.19 (−29.58, 27.20)	**RT**		
−18.86 (−67.16, 29.44)	−8.35 (−54.05, 37.35)	−7.16 (−59.65, 45.34)	**CT**	
−**20.55 (−37.20, −3.91)**	−10.04 (−23.60, 3.52)	−8.85 (−33.79, 16.09)	−1.69 (−47.89, 44.50)	**CON**
HDL, mg/dL				
HIIT				
3.51 (−4.53, 11.56)	**RT**			
6.11 (−0.69, 12.92)	2.60 (−3.54, 8.74)	**CT**		
6.81 (0.43, 13.19)	3.30 (−2.12, 8.71)	0.69 (−3.84, 5.23)	**AE**	
**8.00 (2.18, 13.82)**	4.49 (−0.19, 9.16)	1.88 (−2.01, 5.78)	1.19 (−1.64, 4.02)	**CON**
LDL, mg/dL				
HIIT				
−5.03 (−12.52, 2.46)	**RT**			
−7.01 (−15.05, 1.03)	−1.98 (−8.70, 4.75)	**AE**		
−9.64 (−32.68, 13.40)	−4.61 (−27.22, 18.00)	−2.63 (−24.84, 19.58)	**CT**	
−**8.67 (−14.82, −2.53)**	−3.64 (−7.92, 0.64)	−1.66 (−6.85, 3.52)	0.97 (−21.24, 23.17)	**CON**
VO_2_ max, mL/kg·min				
HIIT				
3.06 (−0.05, 6.17)	**AE**			
3.83 (−0.42, 8.09)	0.77 (−2.67, 4.22)	**CT**		
4.25 (0.74, 7.76)	1.19 (−1.52, 3.90)	0.41 (−3.52, 4.35)	**RT**	
**7.41 (4.37, 10.45)**	4.35 (2.61, 6.10)	3.58 (0.60, 6.55)	3.16 (0.59, 5.73)	**CON**

The league table demonstrates the relative efficacy for each pair of comparison showing the effect of exercise type in each row compared with exercise type in each column. Data are presented as mean difference (95% CI). CON, control group; AE, aerobic exercise; RT, resistance training; CT, aerobic combined resistance training; HIIT, high-intensity interval training. BW, body weight; BMI, body mass index; WC, waist circumference; %BF, percentage body fat; FBG, fasting blood glucose; FINS, fasting blood insulin; TG, triglycerides; HDL, high-density lipoprotein; LDL, low-density lipoprotein. The most effective exercise for each indicator was highlighted in bolded form

**Figure 2:**
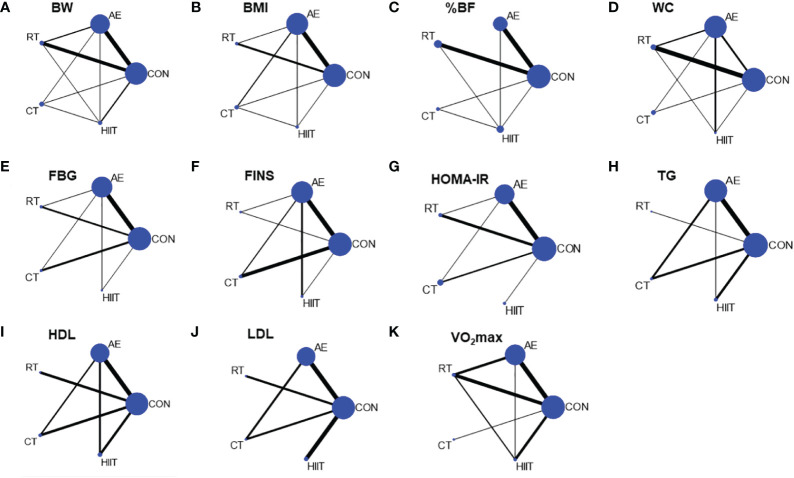
Network diagram of network meta-analysis comparisons. **(A)** BW, body weight, **(B)** BMI, body mass index, **(C)** %BF, percentage body fat, **(D)** WC, waist circumference, **(E)** FBG, fasting blood glucose, **(F)** FINS, fasting blood insulin, **(G)** HOMA-IR, **(H)** TG, triglycerides, **(I)** HDL, high-density lipoprotein, **(J)** LDL, low-density lipoprotein, **(K)** VO_2_ max. CON, control group; AE, aerobic exercise; RT, resistance training; CT, aerobic combined resistance training; HIIT, high-intensity interval training. The size of the nodes represents the number of participants in an intervention, and the thickness of lines between interventions represents the number of studies that compare them.

**Table 3:** Ranking of exercise interventions in order of effective.

Exercise	BW		BMI		WC		%BF		FBG		FINS	
	SUCRA	Mean rank	SUCRA	Mean rank	SUCRA	Mean rank	SUCRA	Mean rank	SUCRA	Mean rank	SUCRA	Mean rank
CON	13.3	4.5	26.3	3.9	0.4	5.0	4.0	4.8	11.2	4.6	5.0	4.8
AE	73.4	2.1	82.4	1.7	39.1	3.4	62.0	2.5	61.1	2.6	57.1	2.7
RT	24.9	4.0	13.1	4.5	43.1	3.3	60.9	2.6	25.8	4.0	42.9	3.3
CT	61.9	2.5	72.5	2.1	86.2	1.6	31.0	3.8	53.6	2.9	53.1	2.9
HIIT	76.5	1.9	55.6	2.8	81.2	1.8	92.2	1.3	98.3	1.1	91.9	1.3
Exercise	HOMA-IR		TG		HDL		LDL			VO_2_ max		
	SUCRA	Mean rank	SUCRA	Mean rank	SUCRA	Mean rank	SUCRA	Mean rank		SUCRA	Mean rank	
CON	16.9	4.3	20.0	4.2	10.4	4.6	20.7	4.2		0.5	5.0	
AE	64.4	2.4	55.6	2.8	32.9	3.7	41.8	3.3		62.2	2.5	
RT	42.3	3.3	51.5	2.9	71.5	2.1	59.8	2.6		40.1	3.4	
CT	28.2	3.9	37.3	3.5	41.9	3.3	36.6	3.5		49.0	3.0	
HIIT	98.1	1.1	85.5	1.6	93.3	1.3	91.2	1.4		98.2	1.1	

CON, control group; AE, aerobic exercise; RT, resistance training; CT, aerobic combined resistance training; HIIT, high-intensity interval training.

BW, body weight; BMI, body mass index; WC, waist circumference; %BF, percentage body fat; FBG, fasting blood glucose; FINS, fasting blood insulin; TG, triglycerides; HDL, high-density lipoprotein; LDL, low-density lipoprotein.

SUCRA, surface under the cumulative ranking.

#### 3.4.1 Anthropometric and body composition

A total of 22 studies assessed BW and 19 studies assessed BMI. The results of NMA showed that only the AE groups had shown a significantly reduced body weight (−2.35 [−4.05, −0.64]) and BMI (−0.9 [−1.38, −0.42]) compared with control. Moreover, 11 studies assessed WC. The CT (−5.94 [−8.33, −3.55]), HIIT (−5.93 [−10.71, −1.15]), RT (−3.04 [−5.42, −0.67]), and AE (−2.85 [−4.63, −1.08]) demonstrated significantly meaningful reductions in WC compared to the control group (**Table 2**). CT was the best option for WC reduction (SUCRA = 86.2). The %BF was assessed in 12 studies. HIIT (−3.93 [−5.73, −2.12]), AE (−2.67 [−4.67, −0.67]), and RT (−2.64 [−4.23, −1.06]) resulted in greater changes in %BF than control. HIIT was regarded as the best exercise training for reducing %BF (SUCRA = 92.2) (**Table 3**). Subgroup analysis showed that short-term exercise could only reduce the %BF (−4.59 [−8.42, −0.76]) while long-term duration of exercise could efficiently reduce body weight, BMI, waist circumference, and %BF. In addition, all exercise could improve anthropometric outcomes for individuals with overweight and obesity (**Supplementary Table 3**).

#### 3.4.2 Glucose metabolism

The FBG, FINS, and HOMA-IR were reported in 15, 15, and 11 studies. AE (−5.12 [−8.73, −1.51]) and HIIT (−14.31 [−22.47, −6.16]) displayed improvements in FBG relative to control. AE (−2.77 [−4.67, −0.87]) and HIIT (−5.94 [−10.48, −1.40]) resulted in greater reductions in FINS relative to control. Regarding HOMA-IR, only the HIIT (−3.01 [−5.08, −0.94]) showed a marked reduction for HOMA-IR compared with control (**Table 2**). According to the SUCRA score, HIIT is probably the best for reducing fasting glucose levels (SUCRA = 98.3), fasting insulin levels (SUCRA = 91.9), and HOMA-IR (SUCRA = 98.1) compared with other exercise types (**Table 3**). The subgroup analysis showed that short-term exercise could just reduce insulin levels (−2.19 [−3.12, −1.26]) while long-term exercise could improve insulin resistance by reducing both insulin levels (−2.53 [−4.34, −0.73]) and HOMA-IR (−0.77 [−1.42, −0.12]) (**Supplementary Table 3**).

#### 3.4.3 Lipid profile

The TC, LDL, HDL, and TG were included in 10, 10, 11, and 8 studies, respectively. Overall, only HIIT elicited meaningful alterations in TC (−16.29 [−30.62, −1.97]), LDL (−8.67 [−14.82, −2.53]), HDL (8.00 [2.18, 13.82]), and TG (−20.55 ([−7.20, −3.91]) and HIIT was the most favorable intervention for improving lipid metabolism (**Tables 2**, **3**). Subgroup analysis showed that short-term exercise had already changed the lipid profiles by reducing TG and LDL levels and increasing HDL levels. The improvement of lipid metabolism was observed in individuals with overweight but not in individuals with obesity (**Supplementary Table 3**).

#### 3.4.4 Cardiovascular function

SBP, DBP, and VO_2_ max were reported in 8, 9, and 13 studies. No meaningful reductions in SBP and DBP were observed in the network comparison. Regarding VO_2_ max, AE (4.35 [2.61, 6.10]), RT (3.16 [0.59, 5.73]), CT (3.58 [0.60, 6.55]), and HIIT (7.41 [4.37, 10.45]) were the exercise modes increasing VO_2_ max (**Table 2**). HIIT (SUCRA = 98.2) ranked the highest (**Table 3**). The results of subgroup analysis showed that short-term exercise has begun to increase VO_2_ max (7.87 [4.92, 10.82]). Both individuals with overweight (6.25 [4.12, 8.37]) and obesity (3.24 [1.89, 4.59]) could gain benefits in increasing VO_2_ max. However, we did not observe any difference in blood pressure in subgroup analysis (**Supplementary Table 3**).

## 4 Discussion

This NMA comprehensively analyzed the current studies about exercise interventions for people with overweight and obesity. Our main findings indicate that AE is probably the best choice for reducing body weight and BMI, while HIIT may be the optimum strategy to improve glucose homeostasis, lipid metabolism, and cardiorespiratory fitness.

### 4.1 Anthropometric and body composition

BW and BMI are often the main endpoints in evaluating obesity. BMI could effectively predict overall mortality but limit the alterations in body composition (45, 46). Therefore, %BF may be considered as a more significant indicator for reflecting the changes in body composition (47). Waist circumference represents the level of visceral fat, which is independently related to all-cause mortality and is considered as a better outcome for predicting the conditions of obesity (48, 49). Our study showed that AE is probably the best in controlling BW and BMI, whereas HIIT could be the optimal exercise intervention for reducing WC and %BF in individuals with overweight and obesity. A recent NMA has shown that CT is the best intervention for reducing WC and %BF with a small reduction in weight loss, but this study did not assess the benefits of HIIT in people with obesity (50). A systematic review comparing HIIT with continuous moderate-intensity training on body composition in adults with overweight and obesity has found that HIIT has similar effects on %BF reduction, but requires ~40% less time commitment (51). Another meta-analysis has reported that HIIT provided nearly 30% more reductions in fat mass than continuous moderate-intensity training (52). The subgroup analysis has found that more than 9 weeks of exercise could effectively improve anthropometric outcomes, and individuals with overweight and obesity could obtain benefits from exercise. In general, our results demonstrated that compared with other types of exercise, AE and HIIT were superior in improving body composition.

### 4.2 Glucose metabolism

Individuals with overweight or obesity are often associated with poor glycemic control, which increase their risk of developing diabetes (53). Our study has shown that HIIT could be the most effective exercise mode in reducing fasting blood glucose, insulin, and HOMA-IR and exercise is more effective in people with obesity (BMI > 30). This may be because overweight participants had relatively healthy glucose metabolism than people with obesity. Additionally, although previous meta-analysis did not consider the effects of duration of exercise on glucose metabolism, our subgroup analysis has found that 4 to 8 weeks of exercise could lower fasting insulin level.

### 4.3 Lipid profile

Guidelines from the American College of Sports Medicine (ACSM) have recommended that more than 150 min of moderate-intensity exercise per week can effectively reduce blood lipid levels (3). The results of NMA showed that HIIT is the most effective mode in improving TG, LDL, and HDL levels. Previous studies have suggested that exercise could improve the TG and HDL levels, but seldom alter TC levels (54), which is consistent with our results. Additionally, a previous meta-analysis demonstrated that HIIT could improve the blood lipid metabolism for type 2 diabetes mellitus (55). Although patients with overweight and obesity included in our study had no complications of dyslipidemia, the results have shown that HIIT moderately reduced lipid levels within the normal range, which was beneficial for reducing the risk of cardiovascular disease. Subgroup analysis found that TG, LDL, and HDL levels were improved in short-term exercise, indicating that exercise could improve lipid metabolism before obvious body weight reduction. However, the reductions in TG and LDL levels were only observed in individuals with overweight, indicating that exercise alone has a limited effect on improving lipid metabolism in people with obesity.

### 4.4 Cardiovascular function

Blood pressure is a commonly assessed measure related to cardiovascular health (56). Many studies have shown that exercise can lower diastolic and systolic blood pressure in people with hypertension (57). For patients with overweight and obesity, we did not find significant changes in blood pressure in both pairwise meta-analysis and subgroup analysis. This may because the baseline of blood pressure in included participants is normal.

Cardiorespiratory fitness (CRF) has long been disregarded as an important moderator of the negative association between mortality and obesity (58). Although increasing studies demonstrated that CRF has a greater impact on morbidity and mortality than body fat percentage (59), most meta-analyses have continued to focus on anthropometric measurements more than CRF when evaluating the effects of exercise for patients with obesity, but have not included CRF in their analyses. Our study has found that all types of exercise can significantly improve CRF, of which HIIT has the highest probability of elevating CRF, followed by AE. One previous meta-analysis has reported that compared with moderate-intensity continuous training, HIIT leads to greater improvement in CRF in children (60). Moreover, another meta-analysis demonstrated that HIIT significantly increases CRF more than continuous moderate-intensity training in patients with chronic diseases (10). Our subgroup analysis found that short-term exercise is enough to increase the VO_2_ max and the benefits existed in both individuals with overweight and obesity. Overall, HIIT may be the preferred option for improving CRF in patients with overweight and obesity.

Previous studies have mainly focused on the effectiveness of HIIT on CRF; beyond that, our research has found that HIIT is also the optimal choice for improving body composition and glucose and lipid metabolism compared with other exercise modalities. Although HIIT has favorable overall improvements, it may not be suitable for people without training foundation. Considering AE is the best for reducing body weight and BMI, it is necessary to take into account the patient’s physical fitness and personal preferences when health professionals advise on exercise training to improve obesity.

## 5 Strengths and limitations

There are some strengths in this study. First, the included indicators are comprehensive and can represent the systemic metabolic status of patients effectively. Additionally, we used NMA to evaluate and rank the efficacy of interventions by combining direct and indirect evidence obtained from RCTs. Moreover, we performed subgroup analysis using the mean baseline BMI to explore the different efficacy of exercise on people with overweight and obesity and the duration of exercise to explore how long it takes to get metabolic benefits. However, there are several limitations to our study. First, most of the included RCT had high risks in the blinding of participants, which is unlikely to carry out in this kind of intervention, but because the outcomes were all measured objectively, this ROB does not seem to influence the objective outcomes. Second, owing to the limited number of included studies, we did not consider the sequence and intensity of exercise. Third, because of the limited direct comparisons for some interventions, the results should be interpreted cautiously. Fourth, of all patients included, 75% were women. Owing to the limited number of included studies, it is difficult to conduct subgroup analysis of gender, and considering the imbalance of sex ratios, this result may be more suitable for female patients. Fifth, although the majority (46%) of included patients used AE, there was no significant inconsistency in the effects of different exercises, indicating that the difference in number of people did not significantly affect our conclusions.

## 6 Conclusion

In summary, our study has demonstrated that different types of exercise have varying efficacy for different metabolic abnormalities. AE produces the best results in reducing BW and BMI, whereas HIIT is most likely the best exercise intervention for improving body composition and systemic metabolic status in adults with overweight and obesity. The findings display a deeper insight into the exercise prescription for individuals with overweight and obesity in the real world.

## Data availability statement

The original contributions presented in the study are included in the article/**Supplementary Material**. Further inquiries can be directed to the corresponding author.

## Author contributions

HW: Data curation, Methodology, Software, Writing – original draft. RC: Data curation, Writing – review & editing. LX: Data curation, Writing – review & editing. FH: Conceptualization, Funding acquisition, Writing – review & editing.
