# Supplementary material for: Comparative efficacy of exercise training modes on systemic metabolic health in adults with overweight and obesity: a network meta-analysis of randomized controlled trials
Source: Front Endocrinol (Lausanne). 2024 Jan 16;14:1294362. doi: 10.3389/fendo.2023.1294362 (PMC10823366; doi:10.3389/fendo.2023.1294362)
Supplement: Supplementary file 1 [file DataSheet_1.docx]

| **Content** | **Pages** |
| --- | --- |
| Table S1. Search strategy. | 2-4 |
| Table S2. The classification of exercise training. | 5 |
| Table S3. The results of pairwise meta-analysis and subgroup analysis. | 6 |
| Figure S1. Cochrane risk bias evaluation chart. | 7 |
| Figure S2-23. Interval plot and cumulative ranking probability plots of network meta-analysis. | 8-18 |
| Figure S24-34. Local inconsistency of network meta-analysis. | 19-24 |
| Figure S35-45. Funnel plots of network meta-analysis. | 24-29 |

**Table S1.** search strategy for Pubmed, Web of science, Embase and Cochrane.

| Pubmed（139） | ((('aerobic exercise'[Title/Abstract] OR 'endurance exercise'[Title/Abstract] OR 'resistance exercise'[Title/Abstract] OR 'physical activity'[Title/Abstract] OR 'Strength training'[Title/Abstract] OR 'combined training'[Title/Abstract] OR HIIT[Title/Abstract] OR 'High-intensity interval training'[Title/Abstract]) AND (Obesity[Title/Abstract] OR obese[Title/Abstract] OR overweight[Title/Abstract] OR Overnutrition[Title/Abstract])) AND ('body weight'[Title/Abstract] OR BMI[Title/Abstract] OR 'body fat percentage'[Title/Abstract] OR 'waist circumference'[Title/Abstract] OR triglycerides[Title/Abstract] OR cholesterol[Title/Abstract] OR 'high-density lipoprotein'[Title/Abstract] OR 'low-density lipoprotein'[Title/Abstract] OR 'fasting blood glucose'[Title/Abstract] OR 'fasting blood insulin'[Title/Abstract] OR HOMA-IR[Title/Abstract] OR 'systolic blood pressure'[Title/Abstract] OR 'diastolic blood pressure'[Title/Abstract] OR 'VO2 max'[Title/Abstract])) AND ('randomized clinical Trial'[Title/Abstract]) |
| --- | --- |
| Web of science（2516） | #1(TS = ('aerobic exercise' OR 'endurance exercise' OR 'resistance exercise' OR 'physical activity' OR 'Strength training' OR 'combined training' OR HIIT OR 'High-intensity interval training') AND Language: (English) AND Publication Type:(Article)  #2(TS = (Obesity OR obese OR overweight OR Overnutrition) AND Language: (English) AND Publication Type: (Article)  #3(TS = ('body weight' OR BMI OR 'body fat percentage' OR 'waist circumference' OR triglycerides OR cholesterol OR 'high-density lipoprotein' OR 'low-density lipoprotein' OR 'fasting blood glucose' OR 'fasting blood insulin' OR HOMA-IR OR 'systolic blood pressure' OR 'diastolic blood pressure' OR 'VO_2_ max') AND Language: (English) AND Publication Type: (Article)  #4(TS = (randomized OR 'randomized clinical Trial') AND Language: (English) AND Publication Type: (Article)  #4 AND #3 AND #2 AND #1 |
| Embase（1625） | ('aerobic exercise':ab,ti OR 'endurance exercise':ab,ti OR 'resistance exercise':ab,ti OR 'physical activity':ab,ti OR 'strength training':ab,ti OR 'combined training':ab,ti OR hiit:ab,ti OR 'high-intensity interval training':ab,ti) AND (obesity:ab,ti OR obese:ab,ti OR overweight:ab,ti OR overnutrition:ab,ti) AND ('body weight':ab,ti OR bmi:ab,ti OR 'body fat percentage':ab,ti OR 'waist circumference':ab,ti OR triglycerides:ab,ti OR cholesterol:ab,ti OR 'high-density lipoprotein':ab,ti OR 'low-density lipoprotein':ab,ti OR 'fasting blood glucose':ab,ti OR 'fasting blood insulin':ab,ti OR 'homa ir':ab,ti OR 'systolic blood pressure':ab,ti OR 'diastolic blood pressure':ab,ti OR 'vo2 max':ab,ti) AND (randomized:ab,ti OR 'randomized clinical trial':ab,ti) |
| Cochrane（101） | 'aerobic exercise' OR 'endurance exercise' OR 'resistance exercise' OR 'physical activity' OR 'Strength training' OR 'combined training' OR HIIT OR 'High-intensity interval training' in Title Abstract Keyword AND Obesity OR obese OR overweight OR Overnutrition in Title Abstract Keyword AND 'body weight' OR BMI OR 'body fat percentage' OR 'waist circumference' OR triglycerides OR cholesterol OR 'high-density lipoprotein' OR 'low-density lipoprotein' OR 'fasting blood glucose' OR 'fasting blood insulin' OR HOMA-IR OR 'systolic blood pressure' OR 'diastolic blood pressure' OR 'VO2 max' in Title Abstract Keyword AND randomized OR 'randomized clinical Trial' in Title Abstract Keyword |

| Type | Definition |
| --- | --- |
| Aerobic exercise  （AE） | Exercise training designed to improve the efficiency and capacity of the cardiorespiratory system, such as walking, basketball, soccer. |
| Resistance training  (RT) | Exercise training designed to improve the strength, power, endurance, and size of skeletal muscles, such as elastic bands, weight machines (1). |
| Combined aerobic and resistance training  (CT) | A combination of aerobic exercise and resistance training |
| High-intensity interval training  (HIIT) | Exercises training involves repeated short-to-long bouts of rather high intensity exercise interspersed with recovery periods (2). |

**Table S2.** The classification of exercise training.

Reference：

1.Powell KE, Paluch AE, Blair SN. Physical Activity for Health: What Kind? How Much? How Intense? On Top of What? Annu Rev Public Health (2011) 32:349-65. Epub 2010/12/07. doi:10.1146/annurev-publhealth-031210-101151. Buchheit M, Laursen PB.

2.High-Intensity Interval Training, Solutions to the Programming Puzzle: Part I: Cardiopulmonary Emphasis. Sports Med (2013) 43(5):313-38. Epub 2013/03/30. doi:10.1007/s40279-013-0029-x.

| **Analysis** | **Body weight** | | | **Body mass index** | | | **Waist circumference** | | | **Body fat percentage** | | |  |
| --- | --- | --- | --- | --- | --- | --- | --- | --- | --- | --- | --- | --- | --- |
|  | Studies | WMD (95% CI) | *P* value | Studies | WMD (95% CI) | *P* value | Studies | WMD (95% CI) | *P* value | Studies | WMD (95% CI) | *P* value |  |
| **Exercise** | 22 | -0.68 (-1.32, -1.96) | **0.0001** | 19 | -0.68 (-0.91, -0.45) | **<0.00001** | 11 | -4.79 (-6.20, -3.38) | **<0.00001** | 12 | -2.69 (-4.30, -1.08) | **0.001** |  |
| AE | 12 | -2.35 (-4.05, -0.64) | **0.007** | 15 | -0.9 (-1.38, -0.42) | **<0.0001** | 4 | -2.85 (-4.63, -1.08) | **0.002** | 5 | -2.67 (-4.67, -0.67) | **0.009** |  |
| RT | 8 | -0.33 (-2.95, 2.30) | 0.808 | 5 | 0.29 (-0.61, 1.18) | 0.532 | 5 | -3.04 (-5.42, -0.67) | **0.012** | 5 | -2.64 (-4.23, -1.06) | **0.001** |  |
| CT | 4 | -1.89 (-4.27, 0.49) | 0.12 | 4 | -0.76 (-1.73, 0.21) | 0.124 | 2 | -5.94 (-8.33, -3.55) | **<0.0001** | 2 | -1.27 (-3.76, 1.21) | 0.315 |  |
| HIIT | 4 | -2.79 (-6.25, 0.68) | 0.115 | 4 | -0.48 (-1.96, 1.01) | 0.53 | 2 | -5.93 (-10.71,-1.15) | **0.015** | 4 | -3.93 (-5.73, -2.12) | **<0.0001** |  |
| **Duration** |  |  |  |  |  |  |  |  |  |  |  |  |  |
| 4-8w | 6 | -1.89 (-5.83, 2.04) | 0.35 | 6 | -0.48 (-1.69, 0.73) | 0.44 | 1 | -3.70 (-12.35, 4.95) | 0.4 | 3 | -4.59 (-8.42, -0.76) | **0.02** |  |
| ＞9w | 16 | -1.88 (-3.17, -0.58) | **0.005** | 14 | -0.70 (-1.22, -0.18) | **0.008** | 10 | -4.82 (-6.27, -3.36) | **<0.00001** | 9 | -2.03 (-2.99, -1.07) | **<0.0001** |  |
| **BMI** |  |  |  |  |  |  |  |  |  |  |  |  |  |
| 25-30 | 6 | -3.35 (-7.53, 0.83) | 0.12 | 8 | -1.03 (-2.07, -0.00) | **<0.00001** | 2 | -3.65 (-7.05, -0.25) | **0.04** | 5 | -3.17 (-5.94, -0.40) | **0.02** |  |
| ＞30 | 11 | -1.05 (-1.79,-0.31) | **0.005** | 10 | -0.43 (-0.70, -0.16) | **0.002** | 6 | -4.73 (-6.54, -2.92) | **<0.00001** | 4 | -0.32 (-0.89, 0.25) | 0.27 |  |
|  |  |  |  |  |  |  |  |  |  |  |  |  |  |
| **Analysis** | **Total cholesterol** | | | **Low density lipoprotein** | | | **High density lipoprotein** | | | **Triglycerides** | | |  |
|  | Studies | WMD (95% CI) | *P* value | Studies | WMD (95% CI) | *P* value | Studies | WMD (95% CI) | *P* value | Studies | WMD (95% CI) | *P* value |  |
| **Exercise** | 10 | -4.47 (-14.08, 5.13) | 0.36 | 10 | -4.13 (-7.03, -1.22) | **0.005** | 11 | 2.97 (0.66, 5.29) | **0.01** | 8 | -14.90 (-25.96, -3.83) | **0.008** |  |
| AE | 5 | -1.74 (-13.72, 10.24) | 0.776 | 4 | -1.66 (-6.85, 3.52) | 0.53 | 5 | 1.19 (-1.64, 4.02) | 0.41 | 5 | -10.04 (-23.60, 3.52) | 0.147 |  |
| RT | 2 | 5.85 (-11.47, 23.18) | 0.508 | 2 | -3.64 (-7.92, 0.64) | 0.095 | 2 | 4.49 (-0.19, 9.16) | 0.06 | 1 | -8.85 (-33.79, 16.09) | 0.487 |  |
| CT | 2 | 14.66 (-2.81, 32.13) | 0.1 | 2 | 0.97 (-21.24, 23.17) | 0.932 | 2 | 1.88 (-2.01, 5.78) | 0.34 | 1 | -1.69 (-47.89, 44.50) | 0.943 |  |
| HIIT | 4 | -16.29 (-30.62, -1.97) | **0.026** | 3 | -8.67 (-14.82, -2.53) | **0.006** | 3 | 8.00 (2.18, 13.82) | **0.01** | 3 | -20.55 (-37.20, -3.91) | **0.016** |  |
| **Duration** |  |  |  |  |  |  |  |  |  |  |  |  |  |
| 4-8w | 3 | -13.97 (-42.09, 14.16) | 0.33 | 3 | -7.69 (-13.97, -1.42) | **0.02** | 3 | 8.21 (1.23, 15.19) | **0.02** | 1 | -37.14 (-48.31, -25.97) | **<0.00001** |  |
| ＞9w | 7 | 1.51 (-3.55, 6.56) | 0.56 | 4 | -3.15 (-6.43, 0.12) | 0.06 | 4 | 1.79 (0.8, 2.78) | **0.0004** | 6 | -7.90 (-12.61, -3.20) | **0.001** |  |
| **BMI** |  |  |  |  |  |  |  |  |  |  |  |  |  |
| 25-30 | 4 | -7.34 (-25.67, 10.99) | 0.43 | 4 | -4.95 (-8.29, -1.61) | **0.004** | 4 | 4.94 (0.14, 9.73) | **0.04** | 4 | -19.00 (-33.72, -4.28) | **0.01** |  |
| ＞30 | 5 | -3.02 (-9.99, 3.95) | 0.4 | 5 | -2.38 (-8.47, 3.70) | 0.44 | 5 | 1.63 (0.36, 2.91) | **0.01** | 4 | -4.04 (-16.64, 8.56) | 0.53 |  |
|  |  |  |  |  |  |  |  |  |  |  |  |  |  |
| **Analysis** | **Systolic blood pressure** | | | **Diastolic blood pressure** | | | **VO_2_ max** | | |  |  |  |  |
|  | Studies | WMD (95% CI) | *P* value | Studies | WMD (95% CI) | *P* value | Studies | WMD (95% CI) | *P* value |  |  |  |  |
| **Exercise** | 8 | -3.46 (-8.80, 1.88) | 0.20 | 9 | -0.40 (-4.06, 3.27) | 0.83 | 13 | 4.37 (3.09, 5.64) | **<0.0001** |  |  |  |  |
| AE | 5 | -2.87 (-8.96, 3.21) | 0.355 | 5 | 1.40 (-3.14, 5.93) | 0.546 | 8 | 4.35 (2.61, 6.10) | **<0.0001** |  |  |  |  |
| RT | 4 | -4.78 (-11.64, 2.07) | 0.172 | 4 | -3.15 (-8.48, 2.18) | 0.246 | 4 | 3.16 (0.59, 5.73) | **0.016** |  |  |  |  |
| CT | 0 |  |  | 0 |  |  | 2 | 3.58 (0.60, 6.55) | **0.018** |  |  |  |  |
| HIIT | 3 | -2.55 (-10.78, 5.67) | 0.543 | 2 | 1.06 (-6.35, 8.47) | 0.779 | 3 | 7.41 (4.37, 10.45) | **<0.0001** |  |  |  |  |
| **Duration** |  |  |  |  |  |  |  |  |  |  |  |  |  |
| 4-8w | 2 | 0.19 (-6.72, 7.10) | 0.96 | 2 | -1.90 (-10.97, 7.17) | 0.68 | 3 | 7.87 (4.92, 10.82) | **<0.00001** |  |  |  |  |
| ＞9w | 5 | -4.65 (-11.84, 2.53) | 0.2 | 5 | 0.33 (-4.82, 5.48) | 0.9 | 10 | 3.52 (2.24, 4.81) | **<0.00001** |  |  |  |  |
| **BMI** |  |  |  |  |  |  |  |  |  |  |  |  |  |
| 25-30 | 2 | -7.66 (-18.56, 3.25) | 0.17 | 2 | -4.06 (-12.00, 3.87) | 0.32 | 4 | 6.25 (4.12, 8.37) | **<0.00001** |  |  |  |  |
| ＞30 | 5 | -0.37 (-3.33, 2.58) | 0.8 | 4 | 1.49 (-3.28, 6.26) | 0.54 | 6 | 3.24 (1.89, 4.59) | **<0.00001** |  |  |  |  |
|  |  |  |  |  |  |  |  |  |  |  |  |  |  |
| **Analysis** | **Fasting blood glucose** | | | **Fasting insulin** | | | **HOMA-IR** | | |  |  |  |  |
|  | Studies | WMD (95% CI) | *P* value | Studies | WMD (95% CI) | *P* value | Studies | WMD (95% CI) | *P* value |  |  |  |  |
| **Exercise** | 15 | -4.59 (-7.98, -1.19) | **0.008** | 11 | -2.31 (-3.15, -1.47) | **<0.00001** | 11 | -0.95 (-1.71, -0.20) | **0.01** |  |  |  |  |
| AE | 8 | -5.12 (-8.73, -1.51) | **0.005** | 7 | -2.77 (-4.67, -0.87) | **0.004** | 5 | -0.91 (-1.81, 0.00) | 0.05 |  |  |  |  |
| RT | 4 | -1.25 (-6.36, 3.86) | 0.633 | 2 | -2.03 (-4.87, 0.81) | 0.161 | 2 | -0.50 (-1.84, 0.83) | 0.46 |  |  |  |  |
| CT | 2 | -4.66 (-12.90, 3.59) | 0.269 | 2 | -2.70 (-7.01, 1.60) | 0.218 | 2 | -0.16 (-1.60, 1.28) | 0.828 |  |  |  |  |
| HIIT | 3 | -14.31 (-22.47, -6.16) | **0.001** | 2 | -5.94 (-10.48, -1.40) | **0.01** | 1 | -3.01 (-5.08, -0.94) | **0.004** |  |  |  |  |
| **Duration** |  |  |  |  |  |  |  |  |  |  |  |  |  |
| 4-8w | 5 | -5.78 (-14.24, 2.69) | 0.18 | 3 | -2.19 (-3.12, -1.26) | **<0.00001** | 3 | 0.35 (-4.86, 5.57) | 0.89 |  |  |  |  |
| ＞9w | 10 | -4.00 (-8.04, 0.04) | 0.05 | 8 | -2.53 (-4.34, -0.73) | **0.006** | 8 | -0.77 (-1.42, -0.12) | **0.02** |  |  |  |  |
| **BMI** |  |  |  |  |  |  |  |  |  |  |  |  |  |
| 25-30 | 6 | -6.27 (-12.70, 0.15) | 0.06 | 4 | -3.40 (-4.76, -2.03) | **<0.00001** | 4 | -2.41 (-3.73, -1.09) | **0.0004** |  |  |  |  |
| ＞30 | 6 | -2.81 (-4.48, -1.15) | **0.0009** | 5 | -0.80 (-1.75, 0.15) | 0.1 | 5 | -0.22 (-0.58, 0.14) | 0.23 |  |  |  |  |

**Table S3.** The results of pairwise meta-analysis and subgroup analysis.

CON, control group; AE, aerobic exercise; RT, resistance training; CT, aerobic combined resistance training; HIIT, high-intensity interval training.

|  |  |  |  |  |  |  |  |  |
| --- | --- | --- | --- | --- | --- | --- | --- | --- |


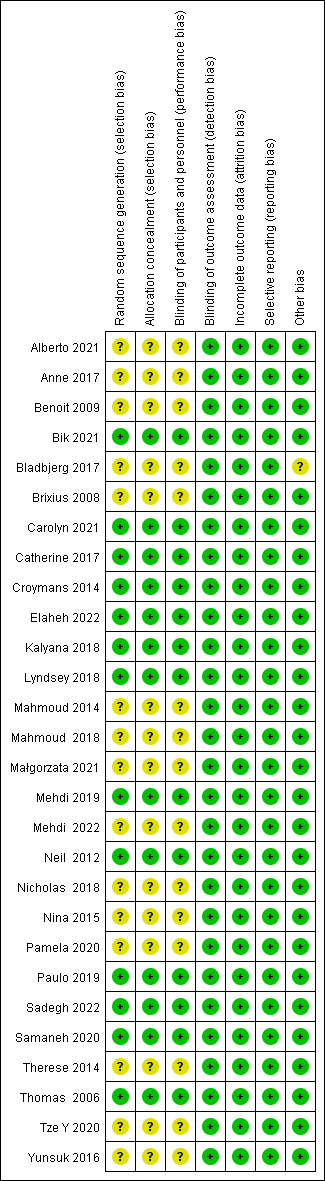


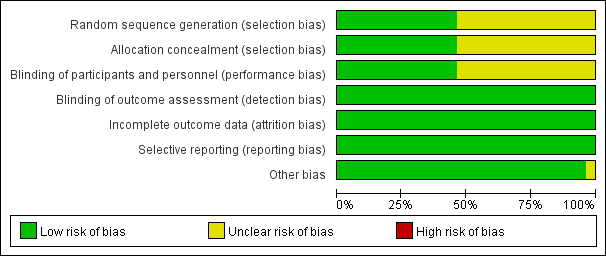


**Figure S1. Cochrane risk bias evaluation chart.**


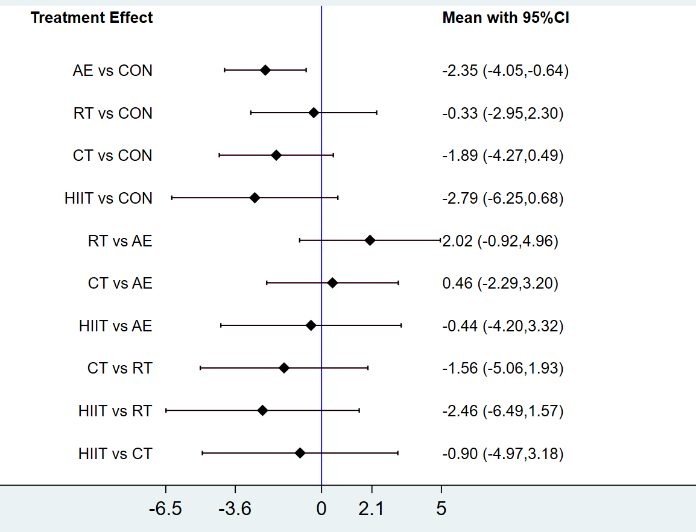


**
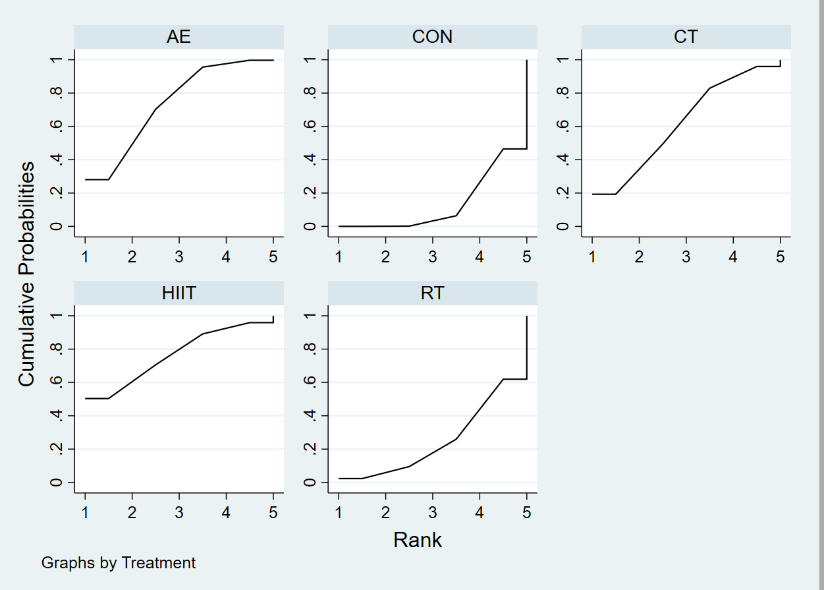
Figure S2.** Interval plot of network meta-analysis for body weight. CON, control group; AE, aerobic exercise; RT, resistance training; CT, aerobic combined resistance training; HIIT, high-intensity interval training.

**Figure S3**. Cumulative ranking probability plots for body weight. CON, control group; AE, aerobic exercise; RT, resistance training; CT, aerobic combined resistance training; HIIT, high-intensity interval training.


**Figure S4**. Interval plot of network meta-analysis for BMI. CON, control group; AE, aerobic exercise;RT, resistance training; CT, aerobic combined resistance training; HIIT, high-intensity interval training.

**Figure S5.** Cumulative ranking probability plots for BMI. CON, control group; AE, aerobic exercise; RT, resistance training; CT, aerobic combined resistance training; HIIT, high-intensity interval training.


**Figure S6.** Interval plot of network meta-analysis for waist circumference. CON, control group; AE, aerobic exercise; RT, resistance training; CT, aerobic combined resistance training; HIIT, high-intensity interval training.

**Figure S7.** Cumulative ranking probability plots for BMI. CON, control group; AE, aerobic exercise; RT, resistance training; CT, aerobic combined resistance training; HIIT, high-intensity interval training.


**Figure S8.** Interval plot of network meta-analysis for percentage body fat. CON, control group; AE, aerobic exercise; RT, resistance training; CT, aerobic combined resistance training; HIIT, high-intensity interval training.

**Figure S9.** Cumulative ranking probability plots for BMI. CON, control group; AE, aerobic exercise; RT, resistance training; CT, aerobic combined resistance training; HIIT, high-intensity interval training.

**Figure S10.** Interval plot of network meta-analysis for FBG. CON, control group; AE, aerobic exercise; RT, resistance training; CT, aerobic combined resistance training; HIIT, high-intensity interval training.

**Figure S11.** Cumulative ranking probability plots for FBG. CON, control group; AE, aerobic exercise; RT, resistance training; CT, aerobic combined resistance training; HIIT, high-intensity interval training.

**Figure S12.** Interval plot of network meta-analysis for FINS. CON, control group; AE, aerobic exercise; RT, resistance training; CT, aerobic combined resistance training; HIIT, high-intensity interval training.

**Figure S13.** Cumulative ranking probability plots for FINS. CON, control group; AE, aerobic exercise; RT, resistance training; CT, aerobic combined resistance training; HIIT, high-intensity interval training.


**Figure S14.** Interval plot of network meta-analysis for HOMA-IR. CON, control group; AE, aerobic exercise; RT, resistance training; CT, aerobic combined resistance training; HIIT, high-intensity interval training.

**Figure S15.** Cumulative ranking probability plots for HOMA-IR. CON, control group; AE, aerobic exercise; RT, resistance training; CT, aerobic combined resistance training; HIIT, high-intensity interval training.

**Figure S16.** Interval plot of network meta-analysis for TG. CON, control group; AE, aerobic exercise; RT, resistance training; CT, aerobic combined resistance training; HIIT, high-intensity interval training.

**Figure S17.** Cumulative ranking probability plots for TG. CON, control group; AE, aerobic exercise; RT, resistance training; CT, aerobic combined resistance training; HIIT, high-intensity interval training.

**Figure S18.** Interval plot of network meta-analysis for HDL. CON, control group; AE, aerobic exercise; RT, resistance training; CT, aerobic combined resistance training; HIIT, high-intensity interval training.

**Figure S19.** Cumulative ranking probability plots for HDL. CON, control group; AE, aerobic exercise; RT, resistance training; CT, aerobic combined resistance training; HIIT, high-intensity interval training.

**Figure S20.** Interval plot of network meta-analysis for LDL. CON, control group; AE, aerobic exercise; RT, resistance training; CT, aerobic combined resistance training; HIIT, high-intensity interval training.

**Figure S21.** Cumulative ranking probability plots for LDL. CON, control group; AE, aerobic exercise; RT, resistance training; CT, aerobic combined resistance training; HIIT, high-intensity interval training.

**Figure S22.** Interval plot of network meta-analysis for VO_2_ max. CON, control group; AE, aerobic exercise; RT, resistance training; CT, aerobic combined resistance training; HIIT, high-intensity interval training.

**Figure S23.** Cumulative ranking probability plots for VO_2_ max. CON, control group; AE, aerobic exercise; RT, resistance training; CT, aerobic combined resistance training; HIIT, high-intensity interval training.

**Figure S24.** Local inconsistency for tumor necrosis body weight.

**Figure S25.** Local inconsistency for BMI.

**Figure S26.** Local inconsistency for waist circumference.

**Figure S27.** Local inconsistency for percentage body fat.

**Figure S28.** Local inconsistency for FBG.

**Figure S29.** Local inconsistency for FINS.

**Figure S30.** Local inconsistency for HOMA-IR.

**Figure S31.** Local inconsistency for TG.

**Figure S32.** Local inconsistency for HDL.

**Figure S33.** Local inconsistency for LDL.

**Figure S34.** Local inconsistency for VO_2_ max.

**Figure S35.** Network meta-analysis funnel plots for body weight.

**Figure S36.** Network meta-analysis funnel plots for BMI.

.

**Figure S37.** Network meta-analysis funnel plots for waist circumference.


**Figure S38.** Network meta-analysis funnel plots for percentage body fat.

**Figure S39.** Network meta-analysis funnel plots for FBG

.

**Figure S40**. Network meta-analysis funnel plots for FBG.

**Figure S41**. Network meta-analysis funnel plots for HOMA-IR.


**Figure S42**. Network meta-analysis funnel plots for TG.

**Figure S43**. Network meta-analysis funnel plots for HDL.

**Figure S44**. Network meta-analysis funnel plots for LDL.

**Figure S45**. Network meta-analysis funnel plots for VO_2_ max.
